# Consumption of foods containing prohibited artificial colors among middle-school children in Nay Pyi Taw union territory, Myanmar

**DOI:** 10.1186/s12889-019-6669-5

**Published:** 2019-03-27

**Authors:** Nwe Oo, Yu Mon Saw, Hnin Nwe Ni Aye, Zaw Zaw Aung, Hnin Nandar Kyaw, Ae Mon Tun, Tetsuyoshi Kariya, Eiko Yamamoto, Nobuyuki Hamajima

**Affiliations:** 1 Department of Food and Drug Administration, Ministry of Health and Sports, Nay Pyi Taw, Myanmar; 2 Department of Health Care Administration, Nagoya University Graduate School of Medicine, Nagoya, Japan; 3 Nagoya University Asian Satellite Campuses Institute, Nagoya, Japan; 4 Mandalay Regional Public Health Department, Ministry of Health and Sports, Mandalay, Myanmar; 5 Department of Public Health, Ministry of Health and Sports, Nay Pyi Taw, Myanmar

**Keywords:** Prohibited foods, Artificial color, Middle school students, Myanmar

## Abstract

**Background:**

Food safety control in Myanmar is regulated by the Department of Food and Drug Administration (FDA). FDA conducts food safety education programs in schools and regular market surveys of foods containing prohibited artificial colors. However, the consumption of foods containing FDA-prohibited artificial colors among school children is understudied. This study aimed to assess the consumption of foods containing FDA-prohibited artificial colors among middle-school children in Nay Pyi Taw Union Territory, Myanmar.

**Methods:**

A cross-sectional study was conducted at eight public schools in Nay Pyi Taw Union Territory in 2017. The schools were selected using simple random sampling with a drawing method. In total, 776 students (359 boys and 417 girls) participated in face-to-face interviews using a structured *questionnaire and photos of foods containing artificial color* published by FDA. A multiple logistic regression was performed to estimate adjusted odds ratio (AOR) for consumption of such foods.

**Results:**

In total, 519 (66.9%) children consumed foods with the FDA-prohibited colors. It was revealed that students at suburban schools were nearly five times more likely to consume foods containing FDA-prohibited *artificial* colors (AOR = 4.84; 95% confidence interval (CI) 2.99–7.82) compared to those at urban schools. In addition, being in the seventh grade (AOR = 3.38; 95% CI 2.30–4.98), availability of prohibited food in school canteen (AOR = 6.16; 95% CI 2.67–14.22), and having a less educated father (AOR = 1.76; 95% CI 1.06–2.92) were positively associated with consumption of the foods with the prohibited colors.

**Conclusion:**

More than half of the students consumed foods with the prohibited colors. Consumption was more frequent among students from suburban schools, those with unsafe foods accessible at their school canteen, seventh graders, and students with a less educated father. The findings highlighted that school food safety programs, which focus on preventing consumption of foods containing FDA-prohibited artificial colors, are *urgently required.* Food safety regulation is also required to ban the sale of unsafe food, especially in school canteens.

**Electronic supplementary material:**

The online version of this article (10.1186/s12889-019-6669-5) contains supplementary material, which is available to authorized users.

## Background

In 2015, the World Health Organization (WHO) estimated that foodborne hazards about 600 million food-borne diseases (FBD) and 420,000 deaths in 2010. The major causes for FBD are parasites, chemicals, and toxins [1]. Artificial food colors are the main source of food toxins. Colors are used during the process of food preparation. Among those colors, there are several chemicals that are not permitted in food preparation. The usage of prohibited artificial food coloring is assessed through post-market surveys regularly in some countries [2]. The usage of prohibited food coloring is strictly controlled in many countries such as the European Council in 1994 and the United Sates’ Food and Drug Administration in 2004 [3].

Artificial food coloring attracts and enhances the appearance of food, and can preserve the color of the original food for a longer duration. Artificial colors are industrial products, which have potential adverse health effects [4]. A meta-analysis study on synthetic food color additives reported that the consumption of artificial colors was controversially associated with attention deficit hyperactivity disorder (ADHD) among children [5]. Moreover, prohibited artificial food colors such as Rhodamine B and Auramine O can cause the genotoxicity and carcinogenicity in both humans and animals. Rhodamine B has effects of carcinogenicity, reproductive and developmental toxicity, neurotoxicity, and acute toxicity in humans [3].

The regulation of food safety in Myanmar is mainly carried out by the Ministry of Health and Sports’ Department of Food and Drug Administration (FDA). The Myanmar Food and Drug Act was first developed in 1928. With the guidance of the World Health Organization’s model for food law, the Myanmar National Food Law was promulgated in 1997. The Myanmar Food and Drug Board of Authority chaired by Minister of Health and Sports is the steering body for food safety policy and guidelines. The Food and Drug Board collaborates on food control measures with other related ministries such as the Customs Department, the Municipal Health Department, and the Consumer Affairs Department [6].

The FDA controls quality and safety of all imported food and local food production. In addition, the FDA also conducts food safety programs for school children, and post-market surveys of school canteens. Laboratory tests of school food are performed annually to determine the presence of prohibited colors, harmful chemical substances, and pathogenic organisms. After completion of post-market surveys, the FDA makes available a list of prohibited foods and photos on its website, and announces these to the public through social media and newspapers. The FDA has reported that the most available prohibited artificial colors in school food were Auramine O, Rhodamine B, and Orange II [7, 8]. Information on the negative health effects of prohibited artificial food colors is also provided by the FDA through food safety education programs in schools across the country.

A United States study reported that the environment of the school influences student’s food choice [9]. Therefore, the school environment is an important factor in the promotion of accessibility to healthy and nutritious food [10, 11]. Although school children may have good knowledge on nutrition and food choice, they are less likely to apply this knowledge in their eating practices. Relevant and precise knowledge plays a critical role in behavior change in eating practices [12].

The Myanmar National Food Law prohibits the production and selling of food that is hazardous and injurious to health [13]. Nonetheless, many home-based food productions in Myanmar apply poor technical knowledge, and some violate the law by producing unsafe food that includes prohibited artificial colors. Food safety is interlinked with the health, trade and technology sectors, and comprehensive regulation can be achieved by a multi-sectoral approach [14]. Although the FDA is strengthening food safety activities through regular post-market surveys and food safety education programs to the community and schools, the consumption of unsafe foods among school children is understudied in Myanmar.

The objective of this study was to assess the consumption of foods containing FDA-prohibited artificial colors among middle-school children in Nay Pyi Taw Union Territory, Myanmar. Middle school in Myanmar’s education system consists of four levels: fifth grade, sixth grade, seventh grade, and eighth grade. Most parents give little money to schoolchildren of young age, who generally carry boxed lunches prepared from home. Therefore, the seventh and eighth grades were chosen for our study.

Nay Pyi Taw is a new Union Territory in development since 2005. According to the 2014 Myanmar population and Housing Census, it had an urban population of 32%, which was nearly the same as the 30:70urban-rural proportion of Myanmar as a whole [15]. Due to its state of being a newly developed Union Territory, the availability of unsafe foods in Nay Pyi Taw school canteens was understudied. To the best of our knowledge, there is no previous study that has determined consumption of foods with FDA-prohibited artificial colors among schoolchildren in Myanmar. Therefore, this study is expected to support development of school food safety policy in Myanmar.

## Methods

### Study subjects

This cross-sectional study was carried out at public schools in Nay Pyi Taw Union Territory from July to September 2017. The data collection was done in eight townships: 1) Zabu Thiri, 2) Pobba Thiri, 3) Dakkhina Thiri, 4) Takone, 5) Leway, 6) Pyinmana, 7) Ottra Thiri, and 8) Zeyar Thiri. Non- proportionate stratified simple random sampling method was used. First of all, public middle and high schools from eight townships were listed and one school from each township was selected by random sampling. From each selected school, 100 students (50 seventh grade students and 50 eighth grade students) were selected by simple random sampling method from school registers, irrespective of the number of students in each school, to get the required sample size. In total, 800 students were interviewed. After removing non-relevant responses and incorrect answers, the total sample size of 776 were considered for further analysis.

All the school canteens inside the compounds of selected schools were listed and assessed with the inspection checklist for selling of FDA-prohibited foodstuffs.

### Data collection

The data were collected through interviews and observations of school canteens using an observational checklist. The students were interviewed face-to-face by our research team using structured questionnaires. The questionnaire was developed by referring to the preventive measures of food safety questions or items developed by WHO, and referring to the pamphlets of school food education program published by the Myanmar FDA. The questionnaire was composed of the following ten sections: 1) socio-demographic characteristics, 2) food safety knowledge including symptoms of FBD, 3) knowledge on mode of transmission, 4) knowledge on hand washing, 5) knowledge on vectors of FBD, 6) knowledge on food storage places, 7) knowledge on reheating of food, 8) knowledge on frozen food, 9) knowledge on artificial food colors, and 10) knowledge on environmental sanitation (Additional file 1). Finally, consumption of FDA-prohibited foodstuffs was assessed by showing the food sample photo in the book “Lists of Prohibited Food” published by FDA, Ministry of Health and Sports, Myanmar. The school canteen was also assessed for the sale of FDA-prohibited foodstuffs.

### Study measure

The independent variables were the socio-demographic characteristics and food safety knowledge of the students. The scores for each knowledge question were given according to the response (e.g., 1 for positive statement and 0 for the negative statement). The score below the mean value was regarded as low knowledge score and the score equal and above the mean value was regarded as high knowledge score. Sale of foods with FDA-prohibited artificial colors in school canteens was assessed applying a nominal scale. The dependent variable was the consumption of foods containing FDA-prohibited artificial colors, assessed with “yes” or “no” question.

### Statistical analysis

The data analysis was conducted using the Statistical Package for Social Science (SPSS) software program version 24 (IBM SPSS Inc.). A multiple logistic regression test was used to estimate the odds ratios (OR) and 95% confidence intervals (CI). A *P*-value less than 0.05 was considered as significant.

## Results

Table 1 shows the background characteristics of student respondents. Most of them (81.3%) were younger than 13 years at the time of the survey. Among 776 students, 383 (49.4%) were from the seventh grade and 393 (50.6%) were from the eighth grade. The majority of their fathers (72.4%) and mothers (64.6%) were educated up to secondary school and above. In occupational status, most of their fathers (67.8%) and mothers (50.6%) had a regular job. Nearly all of the students (95.5%) in this study had never received food safety information from health talks, television, radio, or booklets.

**Table 1:** Background characteristics of middle school children (*N* = 766)

Characteristics	Male (*n* = 359)		Female (*n* = 417)		Total (*N* = 766)	
	n	%	n	%	N	%
Location of school						
Zabuthiri Township	37	10.3	42	10.1	79	10.2
Pobbathiri Township	53	14.8	64	15.3	117	15.1
Ottrathiri Township	51	14.2	49	11.8	100	12.9
Zayarthiri Township	45	12.5	51	12.2	96	12.4
Tatkone Township	41	11.4	57	13.7	98	12.6
Leway Township	48	13.4	55	13.2	103	13.3
Dakkhinathiri Township	50	13.9	49	11.8	99	12.8
Pyinmana Township	34	9.5	50	12.0	84	10.8
Age group						
11-13 years	276	76.9	355	85.1	631	81.3
14-15 years	83	23.1	62	14.9	145	18.7
Grade						
Seventh grade	186	51.8	197	47.2	383	49.4
Eighth grade	173	48.2	220	52.8	393	50.6
Father education						
Primary school and below	108	30.1	106	25.4	214	27.6
Secondary school and above	251	65.9	311	74.6	562	72.4
Mother education						
Primary school and below	128	35.7	145	34.8	27.3	35.2
Secondary school and above	231	64.3	272	65.2	503	64.8
Father occupation						
Irregular job	119	33.1	131	31.4	250	32.2
Regular job	240	66.9	286	68.6	526	67.8
Mother occupation						
Irregular job	162	45.1	221	53.0	383	49.4
Regular job	197	54.9	196	47.0	393	50.6
Ever received food safety information						
No	16	4.5	19	4.6	35	4.5
Yes	343	95.5	398	95.4	741	95.5

Table 2 presents the students’ knowledge on food safety and consumption of FDA-prohibited foods distributed by sex. Most of the students did not know the symptoms of FBD. In total, 37.6% of boys and 39.8% of girls had high knowledge scores on symptoms of FBD. Significantly more girls (40.5%) had high knowledge on mode of transmission of FBD than boys (30.6%). Regarding knowledge on hand washing, vectors of FBD, and food storage condition, the students had a high knowledge score with 64.2, 62.6, and 67.4%, respectively. Most of the students (94.2%) could give the correct response on environmental sanitation.

**Table 2:** Food safety knowledge and consumption of FDA^#^ prohibited artificial color containing foods among middle school children (*N* = 766)

Variables	Male (*n* = 359)		Female (*n* = 417)		Total (*N* = 766)		*P*-value
	n	%	n	%	N	%	
Knowledge on FBD^b^ symptoms							0.56
Low knowledge	224	62.4	251	60.2	475	61.2	
High knowledge	135	37.6	166	39.8	301	38.8	
Knowledge on mode of transmission of FBD^b^							< 0.01
Low knowledge	249	69.4	248	59.5	497	64.0	
High knowledge	110	30.6	169	40.5	279	36.0	
Knowledge on hand washing							0.02
Low knowledge	145	40.4	133	31.9	278	35.8	
High knowledge	214	59.6	284	68.1	498	64.2	
Knowledge on vectors of FBD^b^							0.88
Low knowledge	133	37.0	157	37.6	290	37.4	
High knowledge	226	63.0	260	62.4	486	62.6	
Knowledge on food storage							0.70
Low knowledge	120	33.4	133	31.9	253	32.6	
High knowledge	239	66.6	284	68.1	523	67.4	
Knowledge on environmental sanitation							1.00
Low knowledge	21	5.8	24	5.8	45	5.8	
High knowledge	338	94.2	393	94.2	731	94.2	
Knowledge on reheating of food							0.18
Low knowledge	115	32.0	115	27.6	230	29.6	
High knowledge	244	68.0	302	72.4	546	70.4	
Knowledge on artificial food colors							0.37
Low knowledge	35	9.7	33	7.9	68	8.8	
High knowledge	324	90.3	384	92.1	708	91.2	
Knowledge on frozen food							0.15
Low knowledge	185	51.5	237	56.8	422	54.4	
High knowledge	174	48.5	180	43.2	354	45.6	
Total knowledge score on food safety information							0.61
Low knowledge	159	44.3	177	42.4	336	43.3	
High knowledge	200	55.7	240	57.6	440	56.7	
Consumption of FDA^a^ prohibited foods							0.15
No	109	30.4	148	35.5	257	33.1	
Yes	250	69.6	269	64.5	519	66.9	

*FDA*^a^, Food and Drug Administration; ^b^*FBD*, Food borne diseases

About two-third of the students had high knowledge score on reheating of food. To this question, 72.4% girls and 68.0% boys answered correctly. For knowledge on frozen food, only 45.6% of the students had high scores. The majority of students had high knowledge on artificial food colors. Male students (90.3%) and female students (92.1%) could give the correct response on artificial color-containing food. Irrespective of their knowledge score, two-third of the students (66.9%) consumed foods containing FDA-prohibited artificial colors. Male students (69.6%) were more likely to consume artificial color-containing food than female students (64.5%), although the difference was not significant.

**Fig. 1:**
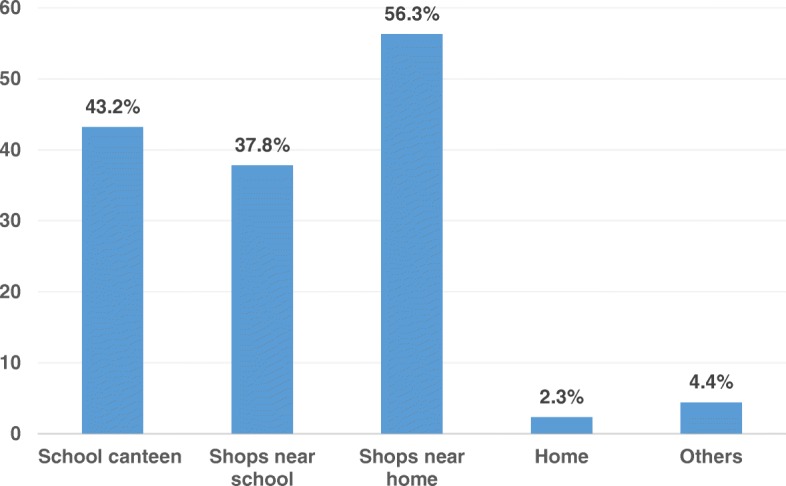
Sources to obtain the Food and Drug Administration prohibited artificial color containing foods among middle school children (*N* = 519)

The Fig. 1 illustrates the sources to obtain foods containing FDA-prohibited artificial colors among middle school children in Nay Pyi Taw, Myanmar. The children accessed foods containing FDA-prohibited artificial colors from their school canteen (43.2%), shops near school (37.8%), shops near home (56.3%), home (2.3%), and other sources (4.4%).

Table 3 shows adjusted OR (AOR) and 95% CI of consumption of foods containing FDA-prohibited artificial color. The students from suburban schools were nearly five times more likely to consume the unsafe foods than students from urban schools; AOR = 4.84 and 95% CI 2.99–7.82. More importantly, the availability of unsafe foods in school canteen was positively associated with the students’ consumption of the foods (AOR = 6.16; 95% CI 2.67–14.22). The consumption was also high among the students with a less educated father (primary school and below) (AOR = 1.76; 95% CI 1.06–2.92), and the seventh graders (AOR = 3.38; 95% CI 2.30–4.98) compared to the eighth graders. On the contrary, the students with a low knowledge score on vectors of FBD were less likely to consume unsafe foods than those with high knowledge score (AOR = 0.58; 95% CI 0.4–0.83).

**Table 3:** Odds ratio (OR) and 95% confidence interval (CI) of consumption of FDA^a^ prohibited artificial color containing foods among middle school children

Characteristics	Unadjusted		Adjusted	
	OR	95%CI	OR	95%CI
Sex				
Girls	1	Reference	1	Reference
Boys	1.26	(0.93–1.71)	1.37	(0.95–1.97)
Age (years)				
11-13 years	1	Reference	1	Reference
14-15 years	1.16	(0.80–1.70)	0.94	(0.59–1.49)
Location of school				
Urban	1	Reference	1	Reference
Sub-urban	8.97	(6.14–13.09)*	4.84	(2.99–7.82)*
Grade				
Eighth grade	1	Reference	1	Reference
Seventh grade	2.53	(1.85–3.45)*	3.38	(2.30–4.98)*
Father education				
Secondary and above	1	Reference	1	Reference
Primary school and below	1.68	(0.91–1.71)	1.76	(1.06–2.92)***
Mother education				
Secondary and above	1	Reference	1	Reference
Primary school and below	1.24	(0.91–1.71)	0.76	(0.48–1.22)
Father occupation				
Regular job	1	Reference	1	Reference
Irregular job	1.20	(0.87–1.66)	1.04	(0.69–1.57)
Mother occupation				
Regular job	1	Reference	1	Reference
Irregular job	0.85	(0.63–1.14)	0.78	(0.49–1.24)
Ever received food safety education				
Yes	1	Reference	1	Reference
No	1.45	(0.67–3.15)	1.22	(0.53–2.80)
Availability of FDA^#^ prohibited foods in school canteen				
No	1	Reference	1	Reference
Yes	18.68	(9.43–37.02)*	6.16	(2.67–14.22)*
Knowledge on mode of transmission				
High knowledge	1	Reference	1	Reference
Low knowledge	0.73	(0.53–0.99)*	0.73	(0.50–1.07)
Knowledge on food color				
High knowledge	1	Reference	1	Reference
Low knowledge	1.04	(0.61–1.77)	0.91	(0.48–1.69)
Knowledge on vectors of food borne diseases				
High knowledge	1	Reference	1	Reference
Low knowledge	0.66	(0.49–0.90)*	0.58	(0.40–0.83)**

*FDA*^a^, Food Drug and Administration; *P < 0.001, **P < 0.01, ***P < 0.05

## Discussion

To the best of our knowledge, this is the first study that examines the consumption of foods containing FDA-prohibited artificial color among middle-school children in Myanmar. This study revealed that the availability of the unsafe foods in the school canteen was positivity associated with students’ consumption of those foods. Similarly, students from suburban schools, students who belonged to the seventh grade, and students with a less educated father were more likely to consume unsafe foods than their counterparts. The students with a lower knowledge score on vectors of FBD were less likely to consume the unsafe foods.

The availability of foods containing FDA-prohibited artificial color at school canteen was a strong predictor of the consumption of such foods in this study. Our study showed that students who could obtain such foods in their school canteen were six times more likely to consume those foods than those who could not. Moreover, foods with artificial colors are visually appealing and attractive to children that leads to consumption among school children. The study from the United States reported that the main factors for food choice by children are their senses of taste and appearance of the food [16].

Food accessibility and availability are also the positive factors for food choices and eating practices among children [9, 17]. In Myanmar, students have to spend most of their time at schools on a day-to-day basis. Students are not allowed to go out during break time and school hours. So, their school environment plays an important role in their food choice. The availability of nutritious food in school lunch had a positive effect on eating behavior of the students of fifth to eighth grade in Philadelphia, Pennsylvania, in the United States [18]. A study in Thailand reported that availability of fruits at home could nearly increase fruit consumption three times among children of fourth to sixth grade [19].

The present study clearly reveals that the school environment plays an important role in the food choice of the students. The majority of students who consumed unsafe foods obtained the foods at their school canteen, shops near school, and shops near home. In Myanmar, school health education programs are delivered by Ministry of Health and Sports, which are a part of disease prevention, environmental sanitation, and food sanitation measures in all schools. Sustainable and integrated teamwork should be applied among Ministry of Health, Ministry of Education, and Parents-Teacher Association to prevent the sale of unsafe foods at school canteens. In addition, the snack shops near school and home should be supervised by the FDA and other related authorities, such as the city development committee, and township police department. A strong and relevant legal framework should be provided for implementation of food safety measures.

This study also showed that higher consumption of foods containing FDA-prohibited artificial color among suburban students than urban students. The students from suburban schools were nearly five times more likely to consume unsafe foods than the students from urban schools. This socioeconomic difference in developing countries can lead to disparities in health outcomes [20]. Another possible reason is that shop owners in urban school canteens might have better knowledge on foods containing FDA-prohibited artificial colors than those in suburban schools. Updated information and facilities in health and educational sectors are more accessible to the urban population. And there may be differences in the marketing efforts of food producers towards suburban stores, as it might be easier to promote foods containing FDA-prohibited artificial color to suburban areas. The supervision activity of school canteen and food store in suburban areas may be less scrutinized by Parent- Teacher Association and local authorities.

The students from urban schools may have more opportunity to obtain health education information via health talks, media, and peer groups than suburban schools [21–23]. Parent-teacher associations in urban schools might be stronger in supervising the school canteen for food safety. This finding complied with the result that urban schools had higher scores than rural schools on “health and nutrition services” by assessing the safe and healthy food and hygienic status of food handlers in school canteens in Lao PDR [24]. This study also suggested that the food safety education should give more attention to the suburban schools. In addition, school canteens in suburban schools need to be supervised more strictly for selling of foods containing FDA-prohibited artificial color.

In this study, seventh grade students were three times more likely to consume foods containing FDA-prohibited artificial color compared to eighth grade students. The eighth grade students might have more opportunity to obtain food safety information, and train for good practices than seventh grade students. Another possible reason is that the lower grade students may not understand the deleterious effects of artificial food colors and are more likely to consume them than higher grade students [25, 26]. Our finding is consistent with a study from the Philippines, which reported that the consumption of unhealthy food was higher among high school students than college students [27]. Food safety education should be initiated in the early stages of education level for better understanding of unhealthy food.

This study also indicated that higher level of paternal education was associated with lower consumption of foods containing FDA-prohibited artificial color. Students whose fathers had lower education were more likely to consume prohibited foods than their counterparts. Children’s eating behavior and food choices are mainly influenced by their parents. Parents can effectively develop children’s experience on food choice and eating pattern [28, 29]. Although our study found an effect by only paternal educational on children’s eating practices, in other studies, maternal education has been shown to have a strong influence on children’s nutritional growth and eating practices [30]. Contrary to our findings, in a China-based study children were more likely to consume unhealthy food of animal fat even though their fathers had higher education [31].

It is notable that students with lower knowledge score on vector of FBD were less likely to consume foods containing FDA-prohibited artificial color than the students with higher knowledge score. This might be due to indirect assessment of knowledge not related with food color. However, there was no significant association between knowledge of food color and consumption of prohibited foods in logistic analysis. The study from China also reported that high knowledge score on food safety was not associated with good practices on food safety [11]. Similarly, food handlers from Ghana could not provide good food safety practices even though they had high food safety knowledge [32].

As a developing country, some imported foods are smuggled due to limited capacity in border area. Moreover, small and medium sized enterprises in Myanmar are challenged by poor technology and low quality raw materials. Therefore, some home based food production cannot fully practice food safety standards. Food safety education training together with technical support should be provided to small industries and home-based food productions to prevent production and selling of unsafe food. Current food safety policy should also be modified to include specific responsibilities to avoid overlapping of duties. Harmonization of food safety measures among different authorities such as health, industries, and local authorities should be strengthened in this policy. It is notable that the Myanmar National Food Law is conceptualized with a farm-to-table approach of comprehensive food safety.

There were some limitations to this study. First, it was only conducted in the central region of Myanmar. The consumption status of foods containing FDA-prohibited artificial food might not be similar with those of the students in other areas of Myanmar. Second, we could not select the school depending on the number of schools in each township, and could select only one school from each township regardless of its number of schools. Third, this study did not measure the food safety knowledge and harmful effects of food color among school children in detail. Further studies including the student’s knowledge on unsafe foods and the harmful effects of FDA-prohibited food color are necessary.

## Conclusions

This study provides a first look at the consumption status of foods containing FDA-prohibited artificial color among middle-school children in Nay Pyi Taw, Myanmar. The study revealed that the availability of unsafe foods at school canteens, students from suburban schools, lower grade students, and lower level of paternal education were important risk factors. Sustainable and targeted intervention programs to prevent consumption of unsafe foods should be implemented in schools and within each community. These intervention programs should be introduced earlier in the lower grade levels and given more attention to suburban schools and students with low socio-economic status, such as a father with a lower education level.

## Additional file

Additional file 1: Questionnaire. (PDF 298 kb)

## Abbreviations

ADHD: Attention Deficit Hyperactivity Disorder
AOR: Adjusted odds ration
CI: Confidence Interval
FBD: Food Borne Diseases
FDA: Food and Drug Administration
OR: Odds Ratio
SPSS: Statistical Package of Social Science
WHO: World Health Organization
