# Supplementary material for: Consumption of foods containing prohibited artificial colors among middle-school children in Nay Pyi Taw union territory, Myanmar
Source: BMC Public Health. 2019 Mar 27;19:344. doi: 10.1186/s12889-019-6669-5 (PMC6437872; doi:10.1186/s12889-019-6669-5)
Supplement: Supplementary file 1 — Questionnaire. (PDF 298 kb) [file 12889_2019_6669_MOESM1_ESM.pdf]

Consumption of foods containing prohibited artificial colors among middle-school children in Nay Pyi Taw Union Territory, Myanmar

This questionnaire is only for research. The result will not be linked to individual respondent and will be kept confidential. Name of the respondent is not needed to describe. It is requested to answer all questions. Thank you for your participation.

Identification Number_____________

Name of the School_______________

Name of interviewer ______________

Date ____________________________

Section 1: Background Characteristics of Middle School Children

1. Age at last birthday (complete year) _______________

2. Sex ( )
   (1) Male
   (2) Female

3. Grade
   (1) Seventh grade
   (2) Eighth grade

4. Father's education level ( )

(1) Illiterate

(2) Can read and write

(3) Primary school level

(4) Secondary school level

(5) High school level

(6) Undergraduate

(7) Graduate

5. Mother's education level ( )

(1) Illiterate

(2) Can read and write

(3) Primary school level

(4) Secondary school level

(5) High school level

(6) Undergraduate

(7) Graduate

6. Father's occupation ( )

(1) Government servant

(2) Agriculture

(3) Livestock breeding

(4) Merchant

(5) Shopkeeper

(6) Odd job

(7) Dependent

(8) Others (Please describe details)__________________

7. Mother's occupation ( )

(1) Government servant

(2) Agriculture

(3) Livestock breeding

(4) Merchant

(5) Shopkeeper

(6) Odd job

(7) Dependent

(8) Others (Please describe details)_________________

8. Have you ever received food safety information before? ( )

(0) No (Skip Question - 9)

(1) Yes

(2) Don't know

(3) No response

9. Where did you get the information about food safety?

(Please answer **"No"** or **"Yes"** for each item.)

(1) Health talk ( )

(2) Television, radio channels ( )

(3) Literature ( )

(4) Peer groups ( )

(5) Others (Please describe details)_________________

Section 2: Knowledge on Symptoms of Food Borne Disease

10. Did you know the health symptoms caused by unsafe food? ( )

(0) No (Skip Question -11)

(1) Yes

(2) Don't know

(3) No response

11. Unsafe food can cause the following conditions;

(Please answer **"No"** or **"Yes"** for each item.)

(1) Abdominal pain ( )

(2) Vomiting ( )

(3) Diarrhea ( )

(4) Chills ( )

Section 3: Knowledge on Mode of Transmission of Food Borne Disease (FBDs)

12. Food borne diseases can be transmitted from the following;

(Please answer **"No"** or **"Yes"** for each item.)

(1) Unclean hands ( )

(2) Washed kitchen wares ( )

(3) Unclean food ( )

(4) Sick person ( )

Section 4: Knowledge on Hand Washing

13. When do you need to wash your hand?
(Please answer **"No"** or **"Yes"** for each item.)

(1) When commencing the class ( )
(2) Before handling food ( )
(3) Before going to toilet ( )
(4) After waste disposal ( )
(5) After going to toilet ( )
(6) When your hands are dirty ( )
(7) Before playing in the ground ( )
(8) After playing with pet ( )

14. What is the best method for proper hand-washing detergent? (Please choose **one** correct answer) ( )

(1) Soap and water
(2) Water only

15. Proper hand washing is needed to wash up to the wrist joint. ( )

(0) No
(1) Yes
(2) Don't know
(3) No response

Section 5: Knowledge on Vectors of Food Borne Disease (FBDs)

16. The following insects can transmit disease to food;
(Please answer **"No"** or **"Yes"** for each item.)

(1) Mosquito ( )
(2) Fly ( )
(3) Cockroach ( )

Section 6: Knowledge on Food Storage

17. The proper way to store the prepared or cooked food is; ( )
(Please choose **one** correct answer)
(1) Normal room temperature
(2) In refrigerator

18. The prepared food should be placed ( )
(Please choose **one** correct answer)
(1) With fly proof cover
(2) Without fly proof cover

19. Raw meat and cooked food should be stored altogether. ( )
(0) No
(1) Yes
(2) Don't know
(3) No response

Section 8: Knowledge on Reheating of Food

20. To prevent food poisoning, how long should leftover food be heated? (Please choose **one** correct answer) ( )
(1) Until they are boiling hot
(2) Just until they hot, but not too hot to eat right away
(3) Just until they are at least room temperature
(4) Reheating isn't necessary

Section 9: Knowledge on Frozen Food

21. Chilling or freezing eliminates harmful germs in food; (Please choose **one** correct answer) ( )

    (1) True

    (2) False

Section 10: Knowledge on Artificial Food Colors

22. Artificial food color can cause harmful effect to health. ( )

    (0) No

    (1) Yes

    (2) Don't know

    (3) No response

23. Do you eat the following food or snacks? ( )
    (Please show the **photos of the FDA-prohibited foodstuffs samples**)

    (0) No (Skip Question - 26)

    (1) Yes

24. Where do you get or buy that food/ snacks?
    (Please answer **"No"** or **"Yes"** for each item.)

    (1) School canteen ( )

    (2) Shops near school ( )

    (3) Shops near home ( )

    (4) From home ( )

    (5) Others (Please describe details) ____________________________

Section 10: Knowledge on Environmental Sanitation

25. Environmental sanitation is important for food safety.

(0) No ( )

(1) Yes

26. Do you think that kitchen dust bins should be provided with cover?

(0) No ( )

(1) Yes

Check lists for inspection of school canteen

No	Items	Findings
1.	Wearing of disposable glove	( )
2.	Soap and water for hand washing	( )
3.	Fly proof food storage	( )
4.	Storage of raw and cooked food separately	( )
5.	Tissue paper for use of consumers	( )
6.	Sanitary water for cooking meal	( )
7.	Bottled drinking water	( )
8.	Fly proof bin	( )
9.	Selling of bright colored food	( )
10.	Selling of FDA prohibited food	( )
